# Evaluation of a Therapeutic Drug Monitoring Strategy for Adalimumab in Psoriasis: A Prospective Pharmacokinetic‐Pharmacodynamic Study

**DOI:** 10.1111/cts.70563

**Published:** 2026-04-30

**Authors:** Shan Pan, Teresa Tsakok, Ruoheng Wei, Nick Dand, Floris C. Loeff, Karien Bloem, Annick de Vries, David Baudry, Michael Duckworth, Angela Pushpa‐Rajah, Alice Russell, Ali Alsharqi, Gabrielle Becher, Ruth Murphy, Shyamal Wahie, Andrew Wright, Christopher E. M. Griffiths, Nick J. Reynolds, Jonathan Barker, Richard B. Warren, A. David Burden, Theo Rispens, Satveer K. Mahil, Joseph F. Standing, Catherine H. Smith, the BADBIR Study Group, the BSTOP Study Group and the PSORT Consortium, Marilyn Benham, Sagair Hussain, Brian Kirby, Linda Lawson, Kathleen McElhone, Anthony Ormerod, Caroline Owen, Michael R. Barnes, Paola Di Meglio, Richard Emsley, Andrea Evans, Katherine Payne

**Affiliations:** ^1^ St John's Institute of Dermatology Guy's and St Thomas' NHS Foundation Trust London UK; ^2^ St John's Institute of Dermatology, School of Basic & Medical Biosciences, Faculty of Life Sciences & Medicine King's College London London UK; ^3^ Department of Medical and Molecular Genetics, School of Basic & Medical Biosciences, Faculty of Life Sciences & Medicine King's College London London UK; ^4^ Sanquin Diagnostic Services Amsterdam the Netherlands; ^5^ Dermatology Department Royal Liverpool and Broadgreen University Hospital Trust Liverpool UK; ^6^ West Glasgow Ambulatory Care Hospital Glasgow UK; ^7^ Department of Dermatology Queens Medical Centre, Nottingham University Teaching Hospitals Nottingham UK; ^8^ Dermatology Department University Hospital of North Durham Durham UK; ^9^ Centre for Skin Sciences University of Bradford Bradford UK; ^10^ Centre for Dermatology Research University of Manchester Manchester UK; ^11^ Department of Dermatology King's College Hospital, King's College London London UK; ^12^ Institute of Translational and Clinical Medicine, Medical School Newcastle University Newcastle upon Tyne UK; ^13^ Department of Dermatology Royal Victoria Infirmary and NIHR Newcastle Biomedical Research Centre, Newcastle Hospitals NHS Foundation Trust Newcastle upon Tyne UK; ^14^ Dermatology Centre Northern Care Alliance NHS Foundation Trust Manchester UK; ^15^ Division of Musculoskeletal and Dermatological Sciences Manchester NIHR Biomedical Research Centre, Manchester Academic Health Science Centre, University of Manchester Manchester UK; ^16^ Institute of Infection, Immunity and Inflammation University of Glasgow Glasgow UK; ^17^ Department of Immunopathology Sanquin Research and Landsteiner Laboratory Amsterdam the Netherlands; ^18^ School of Pharmacy University College London London UK

**Keywords:** adalimumab, pharmacokinetic‐pharmacodynamic, psoriasis, real‐world, therapeutic drug monitoring

## Abstract

Using a real‐world psoriasis cohort, we established the pharmacokinetic‐pharmacodynamic (PKPD) relationship for the biologic therapy adalimumab and evaluated the clinical utility and cost‐effectiveness of a proactive therapeutic drug monitoring (TDM) strategy. A total of 543 patients on adalimumab monotherapy for psoriasis provided 946 pharmacokinetic samples and 1700 Psoriasis Area Severity Index (PASI) disease severity measurements. To describe the PASI change over time, a one‐compartment linear PK model with first‐order absorption and elimination was linked to a turnover mechanism of skin lesions. Based on the PKPD relationship and a predefined therapeutic range, real‐time stochastic simulation was performed for a proactive TDM strategy, where trough levels guided dose escalation or reduction. Compared to the standard care, the TDM strategy improved PASI90 and PASI75 by 37.5% and 12.8%, respectively, with a 25.9% increase in drug costs. In the future, incorporating the PKPD model into a Bayesian therapeutic monitoring algorithm could facilitate individualized adalimumab dosing.

**Study Highlights**

**What is the Current Knowledge on the Topic?**

Serum adalimumab levels are associated with clinical response in patients with moderate‐to‐severe psoriasis. A therapeutic range of 3.2–7.0 μg/mL at trough (or at 2 weeks after drug admin) has previously been defined using the conservative clinical endpoint of PASI75. However, the relevance of this range for optimizing treatment toward the higher efficacy benchmark of PASI90 has not yet been evaluated.

**What Question Did This Study Address?**

Can we evaluate the clinical utility and cost‐effectiveness of therapeutic drug monitoring (TDM) for adalimumab in psoriasis using population pharmacokinetic‐pharmacodynamic modeling and simulation, with PASI90 as the efficacy benchmark for treatment optimisation?

**What This Study Adds to Our Knowledge?**

Using a PKPD model describing adalimumab serum concentrations and clinical response, we were able to perform simulations to evaluate a proactive TDM for adalimumab in psoriasis, where the response rates should be improved with an increase in dose costs overall.

**How This Might Change Clinical Pharmacology or Translational Science?**

In future, incorporating the current study into a Bayesian therapeutic monitoring algorithm could facilitate individualized adalimumab dosing.

## 1 Introduction

Biologic therapies have revolutionized the management of immune‐mediated inflammatory diseases (IMIDs), such as psoriasis, rheumatoid arthritis (RA), and inflammatory bowel disease (IBD), making remission an attainable goal. Tumor necrosis factor (TNF) inhibitors remain the cornerstone of biologic treatment. Nonetheless, treatment response varies widely, with many patients showing either no initial treatment benefit (primary failure) or losing efficacy over time (secondary failure) [1, 2]. This variability is partly driven by differences in drug exposure, influenced by factors such as adherence, body weight, and immunogenicity.

Concentration‐effect associations have been described for most therapeutic antibodies, particularly TNF inhibitors, with higher drug levels generally associated with improved response up to a plateau across IBD, RA, and psoriasis. These findings have led to the proposal of therapeutic ranges for TNF inhibitors to support therapeutic drug monitoring (TDM). TDM, which can be both reactive and proactive, aims to optimize individual treatment outcomes while minimizing the side effects of biologics. Reactive TDM is performed following a clinical event like a disease flare to measure serum drug and anti‐drug antibody levels and guide subsequent dose adjustments. In contrast, proactive TDM measures these parameters at predetermined intervals, regardless of disease activity, and adjusts the dose accordingly to keep levels within the target therapeutic ranges. This field is most advanced in IBD, where reactive TDM is becoming a standard practice to support treatment decisions in patients with loss of response. Although a ‘knowledge gap’ has been highlighted for proactive TDM [3], several randomized controlled trials (RCTs) have demonstrated promising results, along with different guidelines recommending proactive TDM for biologic therapies across various IMIDs [3, 4, 5, 6, 7, 8, 9, 10, 11]. Furthermore, a recent clinical practice guideline has recommended proactive TDM for maintenance intravenous infliximab therapy in IBD, RA, and psoriasis [12].

For several years, the fully human TNF inhibitor monoclonal antibody adalimumab has been the top‐selling drug globally and represents the biggest drug spend for the UK's National Health Service (NHS) [13]. Administered subcutaneously, it exhibits moderate bioavailability (64%) and reaches peak concentration within 5 days [14]. In psoriasis, the longstanding dominance of adalimumab as a first‐line biologic is challenged by newer, more predictable but costlier biologics, such as IL23/IL17 inhibitors [15], which have raised expectations from the previous gold standard of a 75% improvement from baseline in the Psoriasis Area and Severity Index (PASI75) to the more stringent outcome of PASI90 [16].

Nevertheless, adalimumab remains clinically relevant because of its established efficacy, long‐term safety profile (> 1 million patient‐years exposure), and the cost savings from biosimilars following patent expiry in 2018–estimated at £150 million annually for the NHS [13]. Consequently, despite limited implementation of routine TDM, interest in optimizing adalimumab therapy in clinical practice is growing. Therefore, we explored whether TDM can improve PASI response rates toward those seen with newer biologics and at what economic cost.

Psoriasis provides an excellent model for evaluating TDM, as temporal treatment effects can be directly seen and measured. In a large real‐world psoriasis cohort, we previously defined a therapeutic range of 3.2–7.0 μg/mL for adalimumab [17], but validating this through an RCT would be costly and time‐consuming. Therefore, as a pragmatic first step, we used population pharmacokinetic‐pharmacodynamic (PKPD) modeling to simulate proactive TDM (through dose optimisation) versus standard of care (no dose modification). In the future, this PKPD model could ultimately underpin a Bayesian dashboard for real‐time response predictions for dosing and therapy guidance.

## 2 Methods

### 2.1 Patient and Data

#### 2.1.1 Ethics Statement

This study followed the 1996 International Conference on Harmonization in Good Clinical Practice (ICH‐GCP) and the 2008 Declaration of Helsinki. Conduct under the PSORT consortium (Psoriasis Stratification to Optimize Relevant Therapy), it drew on two studies: BSTOP [18] (Biomarkers of Systemic Treatment Outcomes in Psoriasis, with approve from the South East London REC 2 Ethics Committee, 11/H0802/7), and PSORT‐Discovery (PSORT‐D, approved by the National Research Ethics Service Committee London—London Bridge, 14/LO/1685). All participants provided written informed consent before enrolment.

#### 2.1.2 Patients and Setting

BSTOP is a UK‐wide prospective, multicenter (*n* = 79) observational study, initiated in 2011 after a 2009 pilot study. It aims to identify important markers predictive of systemic‐therapy outcomes in severe psoriasis. Eligible UK adults from the British Association of Dermatologists Biologics and Immunomodulators Register (BADBIR) [19] were invited to join. Since 2007, BADBIR has recruited over 22,000 patients with dermatologist‐confirmed psoriasis who initiated or switched systemic therapy within 6 months [20]. The registry records demographics, treatments, comorbidities, adverse effects and longitudinal PASI [21]. PSORT‐D, a sub‐study at 11 BSTOP centers, collected samples at specific time points during the first 3 treatment months using the same criteria for inclusion. After this period, about two‐thirds of PSORT‐D patients continued in BSTOP.

Patients receiving adalimumab monotherapy, with at least one serum sample and PASI measurement within 1 year of treatment initiation were included.

#### 2.1.3 Drug Level and Antidrug Antibody Measurements

Samples of venous blood were obtained at clinic visits from June 2009 to December 2016, centrifuged at 2000 *g* for 10 min, and serum aliquots were stored at −80°C. Given the pragmatic real‐world nature of this study, sample collection was not standardized across all patients or time points, and most samples were drawn during routine clinical care, without consideration of the timing relative to drug administration (neither trough nor peak levels were specifically targeted). The date and time of blood collection were documented, and when available, dates and doses of the most recent adalimumab administration (40 mg) were captured. When dosing records were missing, it was assumed to follow the standard biweekly regimen with full adherence. Adalimumab concentrations were measured using an enzyme‐linked immunosorbent assay [22] with a detection limit of 0.01 μg/mL. Antidrug antibodies were quantified via radioimmunoassay [23], with positivity defined as ≥ 30 arbitrary units/mL. Concentrations below the assay's detection limit were replaced with half the detection limit (0.005 μg/mL) for model estimation.

#### 2.1.4 Clinical Outcome Measures

Psoriasis severity was quantified using PASI. PASI90 (primary outcome) and PASI75 (secondary outcome) were defined as 90% and 75% improvement from baseline.

### 2.2 Adalimumab Real‐World Pharmacokinetics/Pharmacodynamics

Using a sequential approach, a population PKPD model was constructed with nonlinear mixed effects modeling in NONMEM (version 7.6) [24]. The pharmacokinetic (PK) component included data from all patients; the pharmacodynamic (PD) component was limited to the patient subset with baseline PASI ≥ 10, representing individuals eligible for biologic therapy due to severe disease exceeding this threshold.

A linear PK model was first developed to describe the time course of serum adalimumab concentration after doses until the recorded date of the last dose (or if unavailable, until the date of the last serum sample). A one‐compartment model with first‐order absorption and elimination described serum adalimumab concentrations, incorporating allometric weight scaling for clearance and volume of distribution, fixed at 0.75 and 1, respectively [25].

Second, a PD turnover model was constructed to characterize the temporal change in PASI score, where serum drug levels were linked to the therapeutic effect through a maximum effect (*E*_max_) model, representing drug inhibition of psoriasis lesion production.

#### 2.2.1 Covariate Selection

Baseline covariates included demographics (age, sex, ethnicity), lifestyle (alcohol consumption and smoking status), anthropometrics (body weight, body mass index, waist circumference), psoriasis‐specific features (disease duration, palmoplantar involvement), and comorbidities (asthma, major depression disorder, diabetes, dyslipidaemia, hypertension, psoriatic arthritis, liver disease). Only covariates recorded in over 10% of patients were tested in power analyses with sufficient data.

Stepwise Covariate Model‐building (SCM) approach [26] was performed by employing forward inclusion (*p* = 0.05) followed by backward elimination (*p* = 0.01). A covariate was statistically significant if it improved model fit with an objective function value (OFV) change over 3.84 during forward selection (*p* < 0.05) or 6.63 (*p* < 0.01) during backward elimination for one degree of freedom, according to the likelihood ratio test [27].

#### 2.2.2 Model Evaluation

Model fit was assessed using likelihood‐based statistics (OFV from NONMEM) and visual predictive checks (VPC) [28]. Diagnostics plots were generated using R (version 4.4.1).

### 2.3 Simulations of Therapeutic Drug Monitoring Versus Standard Care

All simulations (*n* = 1000 per regimen) incorporated between‐subject variability (BSV) and significant PK covariates from the PKPD model.

Under our proactive TDM algorithm (Figure 4), week 5 trough levels < 3.2 μg/mL prompted dose escalation (given weekly) at week 6. Starting at week 17, PASI90 response status and trough drug levels were assessed for individual patients against 7 μg/mL for potential dose reduction (given every 3 weeks) or dose escalation (given weekly).

The TDM cohort was stratified into six subgroups based on week 5 trough level, week 17 PASI90 status and week 17 trough level (Figure 4). Each subgroup corresponded to one of four predefined TDM regimens outlined below.

#### 2.3.1 Standard‐of‐Care (SOC) Regimen

Initially 80 mg, then 40 mg every 2 weeks, to be started 1 week after the initial dose.

#### 2.3.2 TDM Regimen 1

Patients receive a dose every 2 weeks for the full 6 months if the week 5 trough level ≥ 3.2 μg/mL and:

- (Subgroup 3) PASI90 achieved at week 17 with a trough level of ≤ 7 μg/mL, or
- (Subgroup 6) PASI90 not achieved, but the week 17 trough level is > 7 μg/mL.

#### 2.3.3 TDM Regimen 2

Patients start dosing every 2 weeks from week 0 to 5, then switch to weekly dosing from week 6 onwards. This applies if the week 5 trough level is < 3.2 μg/mL. Weekly dosing continues regardless of PASI90 status at week 17 (Subgroups 1 and 2).

#### 2.3.4 TDM Regimen 3

Patients receive dosing every 2 weeks from week 0 to 17, then reduce to every 3 weeks from week 18 onwards. This applies if the week 5 trough level is ≥ 3.2 μg/mL, PASI90 is achieved at week 17, and the week 17 trough level exceeds 7 μg/mL (Subgroup 4).

#### 2.3.5 TDM Regimen 4

Patients receive dosing every 2 weeks from week 0 to 17, then escalate to weekly dosing from week 18 onwards. This applies if the week 5 trough level is ≥ 3.2 μg/mL, PASI90 is not achieved at week 17, and the week 17 trough level is ≤ 7 μg/mL (Subgroup 5).

At 6 months, PASI90 and PASI75 response rates and dose costs were compared between all TDM dose regimens and SOC to evaluate the clinical and economic benefit of the proactive TDM strategy.

## 3 Results

### 3.1 Patients and Data

In this multicenter (*n* = 60) observational study, 544 psoriasis patients on adalimumab monotherapy met the inclusion criteria; 543 provided 946 serum samples and 539 provided 1700 PASI measurements within the first treatment year. 56 serum samples (5.9%) were below the limit of quantification.

PK analysis included drug and anti‐drug antibody levels from all 543 subjects. For the PD model and subsequent simulations, data were restricted to patients with baseline PASI ≥ 10 (367 patients, 1171 PASI observations). PD parameters estimated from the full cohort of 539 patients are presented in Table S1.

Table 1 showed the baseline characteristics were representative of psoriasis patients eligible for biologics [29]: predominantly male (63.2%), severe chronic disease (median PASI 12; median disease duration 21 years), and frequent metabolic comorbidities (median weight 88.7 kg, diabetes 17%, hypertension 23.2%, psoriatic arthritis 23.6%). Most (69.2%) were biologic‐naïve, and none changed dose or dosing interval during the first treatment year.

**TABLE 1:** Summary of baseline variables for all patients (*n* = 544) including demographics, disease characteristics and comorbidity burden.

Variable	Median or percentage	Range	Missing (%)
Gender (male, %)	63.2	—	0
Age (year)	44.3	[17.4, 80.4]	0
Ethnicity (white, %)	89.5	—	0
Weight (kg)	88.7	[42.6, 170]	2.1
BMI (kg/m^2^)	29.7	[16.6, 63]	13.4
Waist (cm)	101	[46, 165]	8.1
Alcohol (yes, %)	81.2	—	2.5
Smoking (yes, %)	56.3	—	2.5
Baseline PASI	12.2	[0, 43.8]	8.1
Disease duration (year)	21	[2, 63]	6.1
Palms/sole involvement (yes, %)	17.2	—	4.5
Inflammatory arthritis (yes, %)	23.6	—	2.3
Biologic‐naïve (yes, %)	69.2	—	0
Comorbidity (yes, %)	10.6/20.3/17/	—	0/0/0/
(AS/MDD/DM/ DLP/HT/LD)	8.6/23.2/8.7		0/0/0
Creatinine (μmol/L)	76	[42, 149]	2.7

*Note:* Median or percentage: median values for continuous variables or percentage for categorical variables, missing (%): the percentage of records for a variable not available.

Abbreviations: AS, asthma; BMI, body mass index; DLP, dyslipidaemia; DM, diabetes mellitus; HT, hypertension; LD, liver disease; MDD, major depressive disorder; PASI, Psoriasis Area and Severity Index.

### 3.2 The Real‐World PKPD Model for Adalimumab in Psoriasis

**FIGURE 1:**
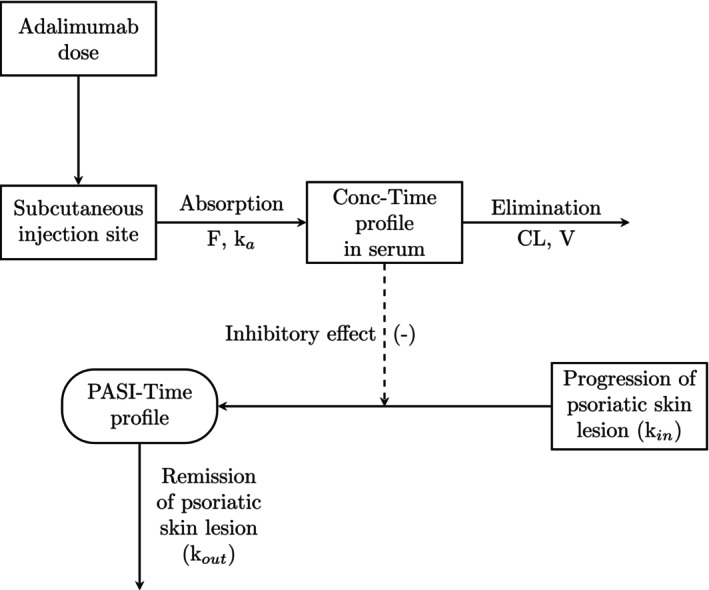
Schematic representation of the final adalimumab population PK–PD model in psoriasis. Adalimumab is administered subcutaneously, absorbed into systemic circulation with first‐order kinetics (rate constant *k*_a_, bioavailability *F*), and eliminated from serum with apparent clearance (CL) and volume of distribution (*V*). The serum concentration–time profile exerts an inhibitory effect on the formation rate (*k*_in_) of psoriatic skin lesions, modeled through an *E*_max_ relationship linking adalimumab concentration to pharmacodynamic response. The Psoriasis Area and Severity Index (PASI)–time profile is described by a turnover process balancing lesion progression (*k*_in_) and remission (*k*_out_). The model captures the dynamic interplay between drug exposure and disease activity over time.

Adalimumab PK was linear [14] and adequately described using a one‐compartment model with first‐order absorption and elimination. PD (PASI evolution in time) was described using a turnover *E*_max_ model, where adalimumab inhibited skin lesion formation. The structure of the final integrated PKPD model is depicted in Figure 1 (NONMEM code for the PK and PKPD models is shown in Supporting Information).

#### 3.2.1 Adalimumab PK

Table 2 summarizes the parameter estimates from the adalimumab PK model. The absorption rate constant and apparent clearance (0.268 day^−1^ and 0.386 L/day) matched published values (0.28 day^−1^ and 0.32 L/day), after the apparent volume of distribution was fixed to 10.8 L as per the reported value [30]. BSV was 32.9% and 76.8% for clearance and volume of distribution, respectively. Relative standard error (RSE) ranged from 3.5% to 35.1%.

**TABLE 2:** Parameter estimates from the adalimumab PK model (*n* = 543).

Parameter (unit)	Estimate	RSE (%)
*k*_a_ (/day)	0.268	11.7
CL/*F* (L/day)	0.386	3.5
*V*/*F* (L)	10.8 [fix]^a^	—
BSV on CL (%)	32.9	17.9
BSV on *V* (%)	76.8	16.5
coeff_weight on CL_	0.75 [fix]^b^	—
coeff_ADA on CL_	0.368	6.6
coeff_female on CL_	0.216	25.1
coeff_waist on CL_	0.888	18.2
coeff_hypertension on CL_	0.177	35.1
coeff_weight on *V*_	1 [fix]^b^	—
Proportional error (%)	19.5	25.9
Additive error (SD)	1.78	8.8

Abbreviations: ADA, anti‐drug antibody; BSV, between‐subject variability; CL/*F*, apparent clearance; coeff, coefficient of a covariate on CL or *V*; corr, correlation coefficient between CL and *V*; *k*_a_, absorption rate constant of rituximab; PK, pharmacokinetic; RSE, relative standard error; SD, standard deviation; *V*/*F*, apparent volume.

^a^ Fixed on the value from Ternant et al. [30].

^b^ Fixed a priori using allometric scaling [25].

Higher apparent clearance correlated majorly with increased body weight, waist circumference, antidrug antibody concentration, female sex and hypertension (all *p* < 0.01); body weight also increased apparent volume of distribution (*p* < 0.01). Allowing the weight exponent on clearance to vary yielded 0.67 (95% CI: 0.35–1.00), confirming a negligible impact on fit and justifying the fixed 0.75 exponent for simplicity and wider applicability.

For missing administration dates, standard two‐weekly dosing (80 mg loading, then 40 mg q2w started 1 week after loading) was assumed. A sensitivity analysis using 349 samples with complete dosing information produced similar clearance (0.358 L/day, 95% CI: 0.319–0.397) to the full model (0.386 L/day, 95% CI: 0.357–0.413), confirming the model's robustness.

**FIGURE 2:**
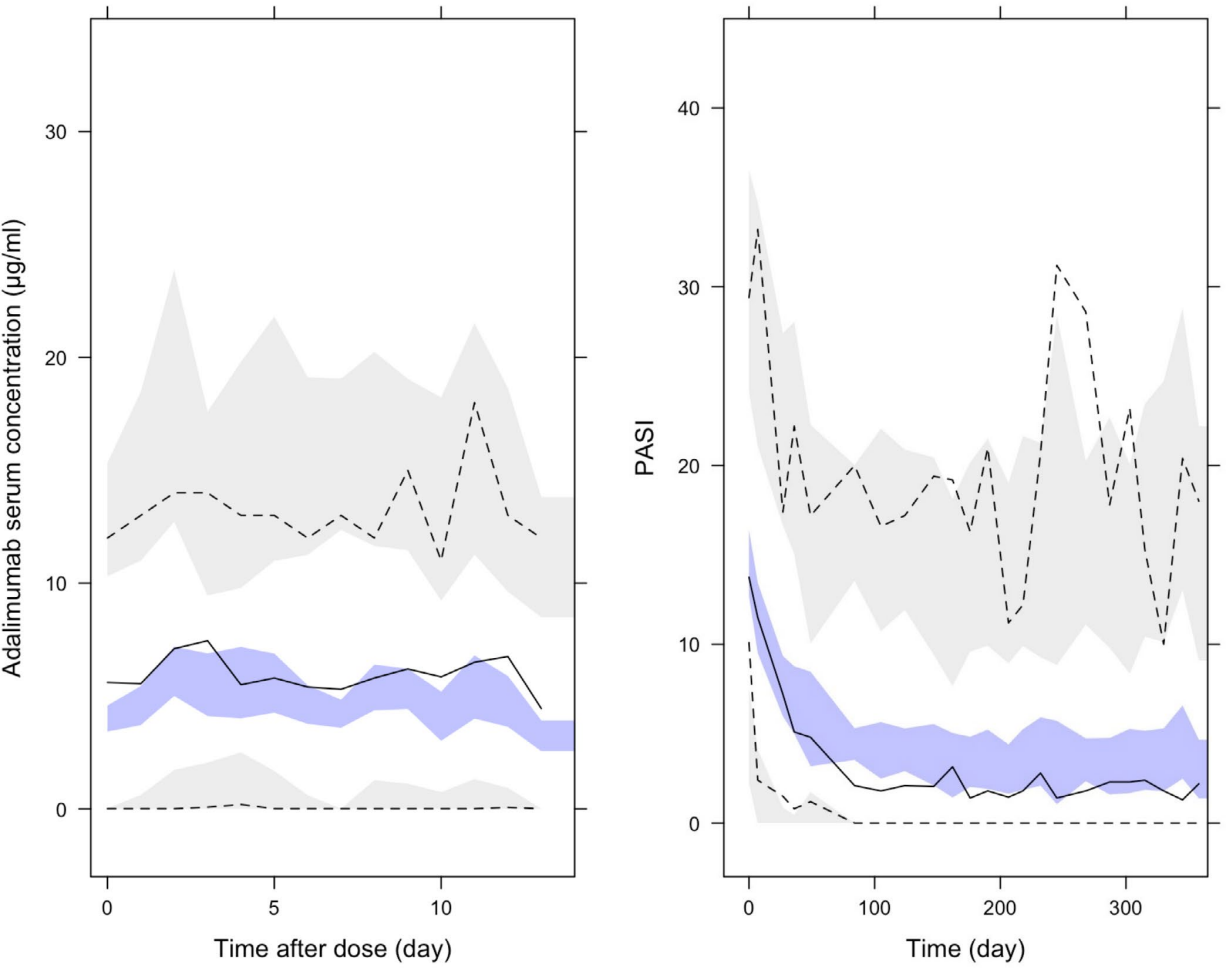
Visual predictive checks (VPCs) for the final adalimumab PK–PD model in psoriasis. Left panel: Observed and simulated serum adalimumab concentrations versus time after dose; right panel: Observed and simulated Psoriasis Area and Severity Index (PASI) versus time on treatment. The solid black line represents the observed median, dashed black lines denote the observed 5th and 95th percentiles, and shaded gray areas indicate the simulated 95% prediction interval. The blue band represents the 95% confidence interval around the simulated median.

The VPC (Figure 2, left panel) demonstrated that the PK model adequately described the time course of serum adalimumab concentrations.

#### 3.2.2 Adalimumab PD

The PD parameter estimates (Table 3) included the lesion turnover half‐life (*k*_out_) of 17.3 days (BSV 116.6%), and EC_50_ (adalimumab concentration resulting in 50% of the maximum inhibition) of 0.95 μg/mL (BSV 97.1%).

**TABLE 3:** Parameter estimates from the adalimumab PD model for patients with baseline PASI ≥ 10 (*n* = 367).

Parameter (unit)	Estimate	RSE (%)
Baseline PASI	14.3	4.6
*k*_out_ (/day)	0.04	7.7
*E*_max_	1 [fix]	—
EC_50_ (μg/mL)	0.95	13.7
BSV on baseline (%)	38.6	16.0
BSV on *k*_out_ (%)	116.6	19.7
BSV on EC_50_ (%)	97.1	22.0
Additive error (SD)	3.2	7.8

Abbreviations: BSV, between‐subject variability; EC_50_, concentration at 50% of maximum inhibition on TNF‐α; *E*_max_, maximum inhibition effect of adalimumab; *k*_out_, elimination rate constant of skin lesions; PASI, Psoriasis Area Severity Index; PD, pharmacodynamic; RSE, relative standard error; SD, standard deviation.

The VPC (Figure 2, right panel) demonstrated similar trends between observed and simulated data, suggesting that the PKPD model adequately captured the temporal evolution of PASI scores.

The goodness‐of‐fit (GOF) diagnostics (Figure S1) for both PK and PD models showed minimal bias. Observations aligned along the line of identity and residuals evenly distributed without time or magnitude‐dependent trends confirmed that both models provided an adequate fit to the data.

### 3.3 Simulations of TDM Versus Standard Care

**FIGURE 3:**
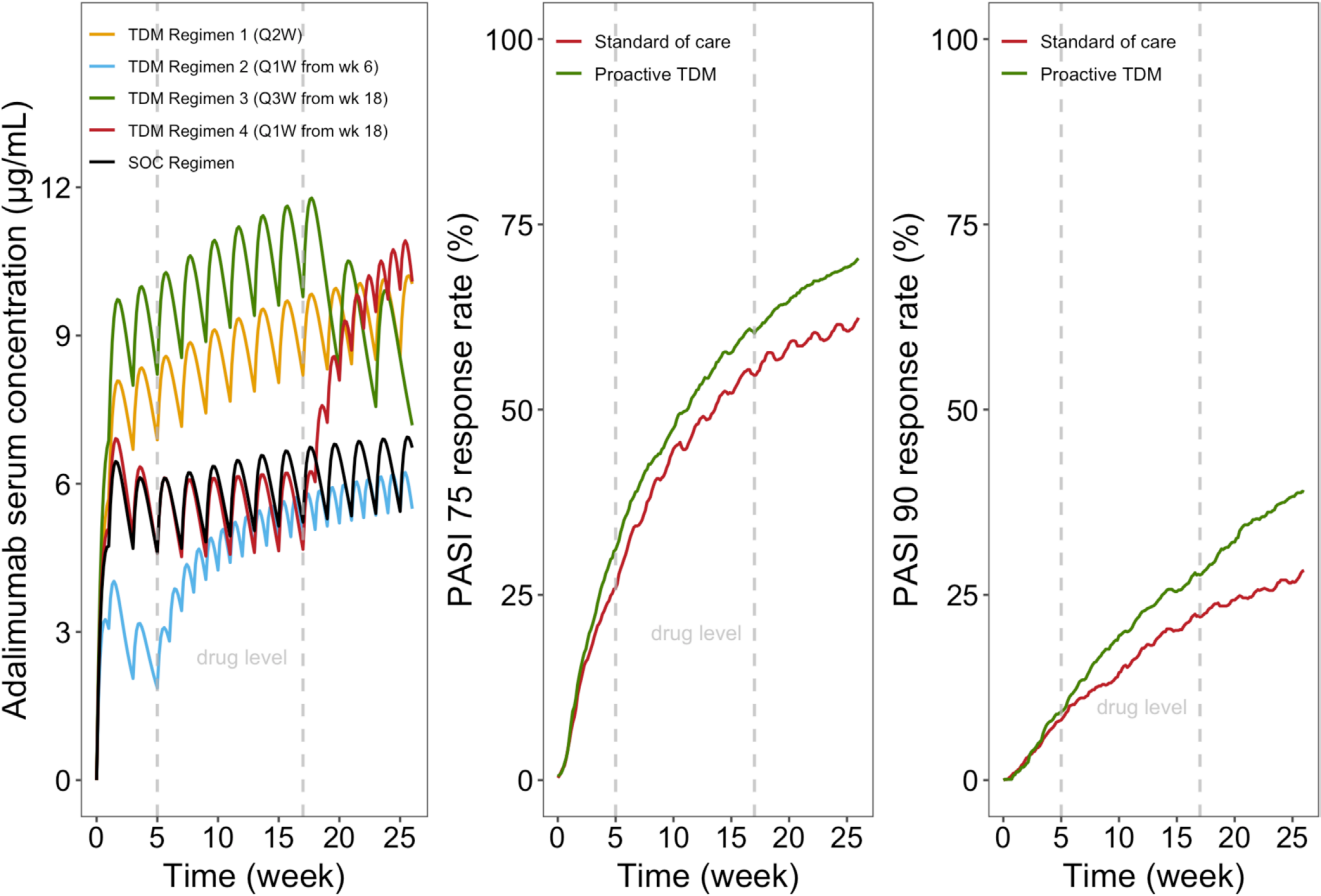
Simulated profiles for the proactive therapeutic drug monitoring (TDM) regimens and the current standard of care (SOC) regimen in psoriasis management. Left panel: Adalimumab serum concentration‐time profiles for SOC (black line) and each of the four TDM dose regimen (orange line: Regimen 1—biweekly throughout 6 months; blue line: Regimen 2—weekly from week 6 for week 5 low trough levels < 3.2 μg/mL; green line: Regimen 3—every 3 weeks from week 18 for week 17 trough levels > 7 μg/mL with PASI90 achieved; red line: Regimen 4—weekly from week 18 for inadequate week 17 trough level ≤ 7 μg/mL with failed PASI90 attainment); Middle panel: Percentage of all simulated patients achieving PASI75 over time (red line: SOC patients; green line: Overall TDM patients); Right panel: Percentage of all simulated patients achieving PASI90 over time (red line: SOC patients; green line: Overall TDM patients). (PASI: Psoriasis Area Severity Index, vertical dotted gray lines: Trough drug level assessed at week 5 and 17 for dose escalation or reduction).

Figure 3 illustrates changes in serum concentration and PASI response over time for the TDM and SOC groups. Before week 5, the population average profiles between the two groups overlapped; after week 5, the TDM group showed higher serum concentration and response rates.

At 6 months, proactive TDM improved PASI90 rates from 28.3% to 38.9% and PASI75 rates from 62.4% to 70.4%, corresponding to 37.5% and 12.8% relative improvements at the cost of a 25.9% increase in total dose.

**FIGURE 4:**
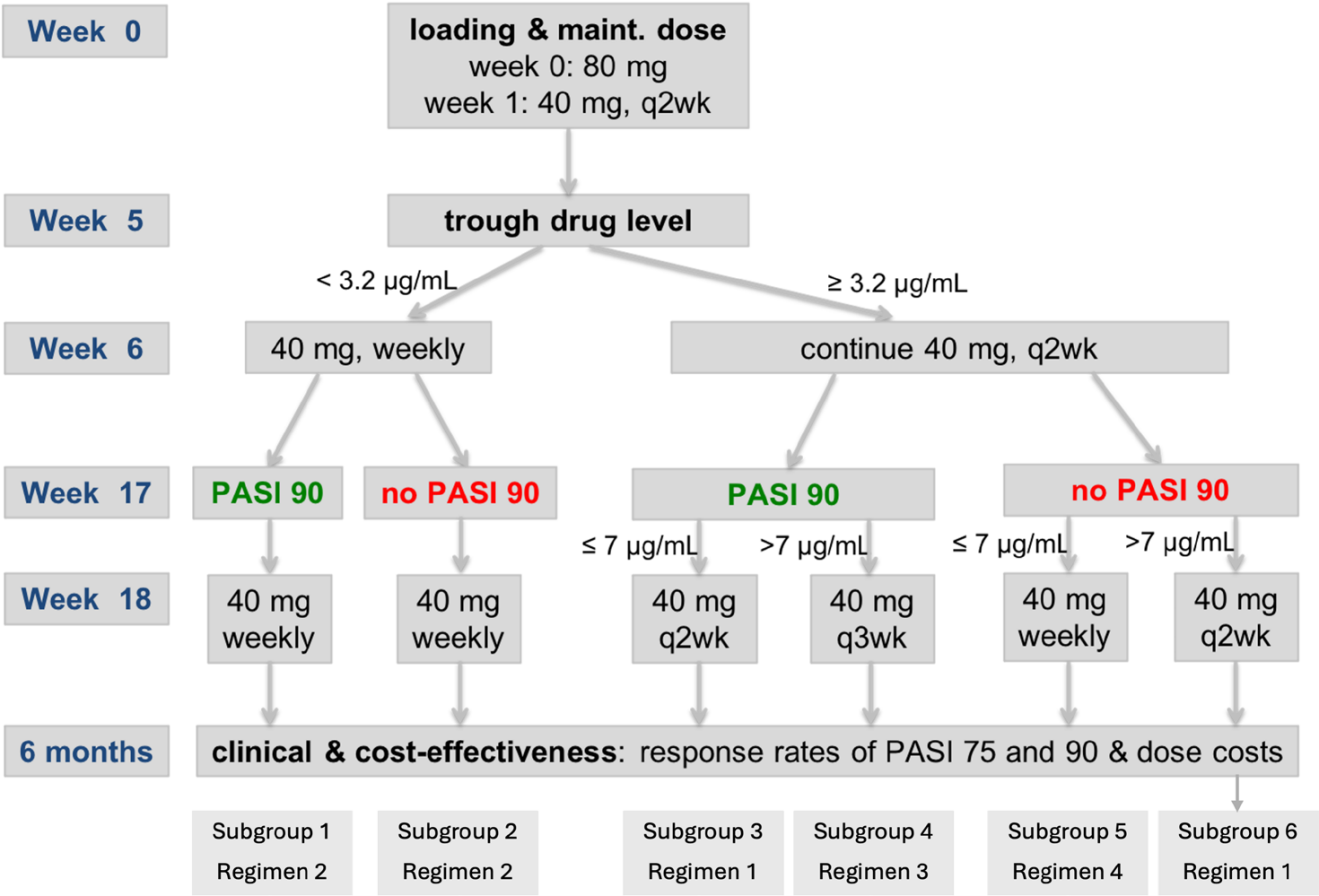
Proactive therapeutic drug monitoring (TDM) algorithm and subgroup definitions for adalimumab in psoriasis. At week 5, trough concentrations < 3.2 μg/mL triggered weekly dosing from week 6 (Regimen 2, forming subgroups 1 and 2 depending on week 17 PASI90 status). At week 17, PASI90 status and trough concentrations > 7 μg/mL guided dose reduction to every 3 weeks (Regimen 3, subgroup 4), whereas trough ≤ 7 μg/mL with no PASI90 led to escalation (Regimen 4, subgroup 5). Biweekly dosing without changes formed Regimen 1, yielding subgroups 3 and 6 according to week 17 PASI90 status and trough level relative to 7 μg/mL. At 6 months, PASI75/90 responses and total dose costs were compared across regimens. (PASI, Psoriasis Area and Severity Index; q2w, every 2 weeks; q3w, every 3 weeks.)

Across the TDM subgroups outlined in the Method (Table S2, Figure 4), 254 patients met the Regimen 1 criteria and required no dose change during treatment, maintaining a biweekly schedule throughout the 6‐month period. Of these, 87 patients (subgroup 3) achieved PASI90 but failed to reach a week 17 trough level above 7 μg/mL, whereas 167 (subgroup 6) failed to achieve PASI90 despite trough levels exceeding 7 μg/mL. A further 345 patients met the Regimen 2 criteria and escalated their doses to weekly from week 6 because their week 5 trough level was below 3.2 μg/mL. Among these, 83 patients achieved PASI90 (subgroup 1) and 262 did not (subgroup 2). An additional 287 patients (subgroup 5) followed Regimen 4, escalating to weekly dosing from week 18 after failing to achieve PASI90 and having a trough level ≤ 7 μg/mL at week 17. Altogether, 31.6% of patients underwent weekly escalation to maintain therapeutic exposure when considering the total simulated population derived from all regimen scenarios. A further 104 patients (subgroup 4) underwent dose reduction to every 3 weeks under Regimen 3 after attaining both PASI90 and trough concentrations > 7 μg/mL at week 17.

At 6 months, PASI90 rates under the TDM strategy were 100%, 7.6%, 100%, 86.5%, 27.5%, and 15.6% across subgroups 1–6, versus 100%, 6.7%, 100%, 100%, 5.4%, and 15.6% under SOC. Corresponding PASI75 rates were 100%, 48.9%, 100%, 100%, 64.8%, and 65.3% under TDM, versus 100%, 39.0%, 100%, 100%, 57.7%, and 65.3% under SOC.

These findings demonstrate that proactive TDM achieved higher overall efficacy than SOC within the same simulated population. Subgroups 2 and 6—those with low early trough levels or persistently subtherapeutic concentrations despite escalation—were least likely to reach PASI90, suggesting that TDM may help identify patients better suited for biologic switching rather than continued escalation.

## 4 Discussion

### 4.1 Key Findings

Within a large‐scale, real‐world psoriasis cohort, a population PK‐PD model was developed for adalimumab and applied to explore the clinical utility of a proactive TDM algorithm incorporating a predefined therapeutic range. Real‐time stochastic simulations suggested that the TDM strategy would improve 6‐month PASI90 and PASI75 response rates compared to standard care, though with overall higher drug costs.

### 4.2 Adalimumab PK Modeling

The PK estimates and the linear behavior of adalimumab were consistent with published data in psoriasis [31], RA [30] and Crohn's disease [32]. The apparent volume of distribution was fixed a priori to a literature value [30] because most serum samples were collected at steady state, limiting estimation due to the flip‐flop phenomenon [33].

This study considered a wider range of covariates than previous adalimumab PK–PD studies, which were constrained with sex, corticosteroid co‐therapy, antidrug antibody, and body weight due to their smaller cohorts [30, 31]. In line with this study, when allometric scaling was added a priori, waist circumference was found to be associated with increased drug clearance. However, we report that clearance increased in females, whereas the RA study found clearance to be higher in men. Patients with hypertension also had higher clearance, likely reflecting underlying obesity, which is known to be associated with hypertension. Higher anti‐drug antibody concentrations were found to significantly increase clearance in both our study and van Huizen et al. [31]. This finding supports the mechanistic understanding that immune complexes are generated between therapeutic and antidrug antibodies, leading to their internalization and subsequent lysosomal degradation [34]. However, the coefficient of antidrug antibody on clearance in our study largely differs from that of van Huizen et al. [31] (0.37 vs. 0.87), likely reflecting that their methotrexate co‐therapy may prevent antidrug antibody formation and lead to lower drug clearance. Future work should assess whether PK‐only models offer comparable predictive performance to PK models with antidrug antibody, as PK testing is more readily available and may support easier clinical implementation.

### 4.3 Adalimumab PD Modeling

Given the chronic and relapsing course of psoriasis, a turnover model captured the dynamic balance between formation and resolution of psoriatic lesions over time, with an estimated lesion turnover half‐life of approximately 2–3 weeks, broadly consistent with studies of both adalimumab and other biologics in psoriasis [31, 35, 36, 37].

Of note, a single parametric distribution best described EC_50_, and we found no significant enhancement in model fit using a mixture model where the patient population was divided into two subgroups. Additionally, no covariates could explain the large variability associated with either EC_50_ or *k*_out_ in the PD model.

### 4.4 Utility of Proactive TDM

Our exploratory simulations suggest that proactive TDM could improve 6‐month response rates through early dose optimisation.

Notably, subgroup 4, which underwent dose reduction from every 2 weeks to every 3 weeks, showed a small decline in PASI90 from 100% to 86.5%, illustrating the model‐driven trade‐off between clinical efficacy and cost; it highlights the need for prospective validation to determine the optimal conditions for safe dose de‐escalation.

Although the therapeutic range (3.2–7.0 μg/mL) was originally based on PASI75 outcomes [17], we used it as a conservative benchmark for PASI90 optimisation, ensuring that any TDM benefits reflect a minimum estimate of its potential.

Our findings of evaluating TDM in psoriasis add to a growing body of evidence in favor of proactive TDM for TNF inhibitors. Particularly, our findings of PASI90 rate improvement may inform the recent clinical practice guideline, which weakly recommends against the use of proactive TDM for patients with IBD, RA, and psoriasis receiving maintenance treatment with adalimumab, due to a low certainty of evidence in previous trial cohorts [12]. Although most studies are retrospective, some notable exceptions exist in the IBD settings. The Trough Concentration Adapted Infliximab Treatment (TAXIT) randomized controlled trial showed that proactive TDM during infliximab's maintenance phase was associated with reduced relapse risk, decreased requirements for rescue therapy, and lower frequency of undetectable drug levels [38]. Furthermore, the Pediatric Crohn's disease Adalimumab‐Level‐based Optimisation Treatment (PAILOT) randomized controlled trial reported that proactive TDM was superior to reactive TDM on the basis of sustained steroid‐free clinical remission [39].

Our TDM strategy was associated with increased dose cost as per the total number of adalimumab doses administered, implying that our cohort of patients more often fit the criteria for dose escalation than for dose reduction. However, upfront costs of additional drug doses and conducting drug‐level assays must be weighed against the potential long‐term cost savings from keeping a higher proportion of patients in clinical remission, where now the drug costs are reduced dramatically, and the cost of drug concentration testing is negligible compared to the total cost of care. Considering the growing implementation of TDM [40], particularly in IBD management and increasingly in psoriasis, studies evaluating the cost‐effectiveness of TDM for TNF inhibitors are few. A 2017 meta‐analysis identified only seven studies across IBD and RA, including two randomized controlled trials and five modeling approaches [41]. Together with a multi‐center observational study in IBD and a more recent systematic review that included 13 studies to analyze the cost‐effectiveness of TDM for TNF inhibitors in IBD [42, 43], the synthesized evidence indicates that TDM results in substantial cost savings with no negative impact on efficacy. However, further prospective validation is clearly needed.

Assuming that decisional algorithms incorporating drug levels are shown to have both clinical and economic utility, a fundamental question remains as to tailoring dose modification to individual patients, that is, when and by how much should we escalate or reduce the dosing regimen? Population PKPD modeling is a first step toward dose individualisation, as a patient's information can be integrated with prior knowledge of the drug's PKPD using Bayesian forecasting [44]. Proof of these concepts has been demonstrated in silico by comparing current TDM algorithms to Bayesian adaptive dosing for infliximab in IBD [45]. Such modeling can then be incorporated into online dashboards, as exemplified by the recent model‐guided risankizumab TDM dashboard for psoriasis [46], to integrate patient factors and drug levels in order to support clinical decision‐making in practice.

### 4.5 Strengths and Limitations

This PKPD study of adalimumab in psoriasis is the largest PKPD study of this drug across any IMID. We draw on real‐world clinical data rather than trial datasets to make our results more representative and generalisable. A major strength lies in our cohort's external validity: over half of all UK psoriasis patients receiving biologics are enrolled on BADBIR, with data contributing 95% of UK dermatology centers prescribing these agents.

Several limitations should be acknowledged. As with most real‐world studies, missing data posed challenges. For absent injection dates, perfect adherence and unchanged dosing were assumed; missing covariates were imputed using medians or modal categories. The lack of a placebo arm precluded assessment of the untreated disease trajectory.

Our pragmatic sampling design led to unbalanced and incomplete PKPD data across patients and time points. Nonetheless, parameter estimates remained consistent with published values [31], suggesting minimal impact on model reliability. Although only 60% of BADBIR centers participated in BSTOP–potentially introducing selection bias–we mitigated this by including all eligible patients with at least one serum sample and PASI measurement, thereby maximizing cohort inclusivity.

Finally, we acknowledge that despite finding large BSV in two PD parameters, namely *k*_out_ and EC_50_, we were unable to identify covariates contributing to this. Furthermore, our TDM algorithm is simple and only used in traditional TDM, which relies on trough‐level serum drug concentrations and a therapeutic range based on our previous work, while the model has utilized pragmatic sampling data, that is, trough and non‐trough. While our exploratory findings require validation in a prospective trial, our study illustrates how proactive TDM can be implemented during induction and maintenance phases to guide dosing decisions, paving the way for more precise, personalized treatment and better outcomes for patients in the future. Looking ahead, extending the current simulation framework beyond 6 months to cover 12–24 months of treatment would enable evaluation of longer‐term immunogenicity dynamics. As antidrug antibodies typically emerge during maintenance therapy, incorporating their temporal effects on clearance could improve the predictive accuracy and clinical applicability of future TDM models.

## Author Contributions

S.P., T.T., R.W., S.K.M., J.F.S., C.H.S., N.D., F.C.L., K.B., A.V., and D.B. wrote the manuscript; T.T., N.D., C.E.M.G., N.J.R., J.B., R.B.W., A.D.B., T.R., C.H.S., and J.F.S. designed research; T.T., N.D., F.C.L., K.B., A.V., D.B., M.D., A.P.‐R., A.R., A.A., G.B., R.M., S.W., A.W., C.E.M.G., N.J.R., J.B., R.B.W., A.D.B., and S.K.M. performed research; S.P., T.T., R.W., S.K.M., J.F.S., C.H.S., N.D., F.C.L., K.B., A.V., C.E.M.G., N.J.R., J.B., R.B.W., and T.R. analyzed the data; S.P., N.D., F.C.L., K.B., A.V., T.R., C.H.S., and J.F.S. contributed new reagents/analytical tools.

## Funding

This work was supported by PSORT, which is in turn funded by a Medical Research Council (MRC) Stratified Medicine award (MR/L011808/1). The Psoriasis Association (RG2/10), the NIHR Biomedical Research Centre at King's College London/Guy's and St Thomas' NHS Foundation Trust, the NIHR Manchester Biomedical Research Centre, and the NIHR Newcastle Biomedical Research Centre. TT is supported by an MRC Clinical Research Training Fellowship (MR/R001839/1). RW is supported by the British Skin Foundation. ND is supported by Health Data Research UK (MR/S003126/1). NJR's research/laboratory is also funded in part by the NIHR Newcastle HealthTech Research Centre in Diagnostic and Technology Evaluation and the NIHR Newcastle Patient Safety Research Collaboration. CEMG, CHS, and NJR are NIHR Senior Investigators. SKM is supported by an NIHR Advanced Fellowship (NIHR302258).

## Conflicts of Interest

C.E.M.G. has received honoraria and/or research grant support (University of Manchester) from AbbVie, Almirall, Bristol Meyers Squibb, Celgene, GSK, Janssen, LEO Foundation, Lilly, Novartis, Pfizer, Sandoz, Sun Pharma, and UCB Pharma. N.J.R. has received honoraria, travel support, and/or research grants (Newcastle University) from AbbVie, Galderma, Novartis, and UCB Pharma Ltd. J.B. has received honoraria, travel support, and/or research grants (King's College) from AbbVie, Pfizer, Novartis, Janssen, Roche, Regeneron, Lilly, UCB, Sun Pharma, Boehringer Ingelheim, and GSK. R.B.W. has received honoraria and/or research grants from AbbVie, Almirall, Amgen, Boehringer Ingelheim, Celgene, Janssen, Leo, Lilly, Novartis, Pfizer, Sanofi, Xenoport, and UCB. A.D.B. has received honoraria from AbbVie, Amgen, Boehringer Ingelheim, Celgene, Janssen, Leo, Lilly, Novartis, and Pfizer. T.R. has received honoraria for lectures from Pfizer, AbbVie, and Regeneron and a research grant from Genmab. D.S. has received departmental research funding from AstraZeneca. C.S. has received departmental research funding from AbbVie, GSK, Pfizer, Novartis, Regeneron, and Roche. N.W. acts as statistician on a trial funded by AstraZeneca. The PSORT consortium has a number of industry partners; see www.psort.org.uk. All other authors declared no competing interests for this work.

## Supporting information


**Figure S1:** Goodness‐of‐fit diagnostics for the final adalimumab PK–PD model. (A) Observed versus individual predictions (IPRED) for adalimumab serum concentrations (left) and PASI response (right). (B) Individual weighted residuals (IWRES) versus time (left) and versus individual predictions (right) for the PK model (top panels) and the PD model (bottom panels). (C) Observed versus population predictions (PRED) for the PASI response. (D) Observed versus population predictions (PRED) for adalimumab serum concentrations. In all panels, blue dots represent observed values. The solid black line denotes the line of identity, and the red line represents a LOESS‐smoothed trend through the data. Dashed black lines in the IWRES plots indicate the ±2 boundaries.

**Figure S2:** Visual Predictive Checks (VPCs) comparison for (Left) the final one‐compartment PK model, (Middle) the two‐compartment PK model using priors from Kang et al. (2020), and (Right) the two‐compartment PK model using priors from Bobadilla et al. (2023). The solid black line represents the observed median, the dashed black lines denote the observed 5th and 95th percentiles, and the shaded gray areas indicate the simulated 95% prediction interval. The blue band represents the 95% confidence interval around the simulated median.

**Figure S3:** Visual Predictive Checks (VPCs) comparison for the PKPD models describing PASI response over time from (Left) the final one‐compartment PK model, (Middle) the two‐compartment PK model using priors from Kang et al. (2020), and (Right) the two‐compartment PK model using priors from Bobadilla et al. (2023). The solid black line represents the observed median, the dashed black lines denote the observed 5th and 95th percentiles, and the shaded gray areas indicate the simulated 95% prediction interval. The blue band represents the 95% confidence interval around the simulated median.

**Table S1:** Parameter estimates from the adalimumab PD model for all patients (*n* = 539).

**Table S2:** Response rates for subgroups after therapeutic drug monitoring (TDM) dosing or standard‐of‐care (SoC) dosing.

**Table S3:** Comparison of PK parameter estimates for adalimumab obtained using the final one‐compartment PK model and two two‐compartment PK models implemented with informative Bayesian priors from Kang et al. (2020) [1] and Bobadilla et al. (2023) [2].

**Table S4:** Comparison of PD parameter estimates from the final one‐compartment PKPD model and those from two‐compartment PK models using Kang et al. and Bobadilla et al. priors; the relative percentage change is calculated relative to the final one‐compartment model.

**Data S1:** Supporting Information.

## Appendix

Members of the BADBIR study group (excluding individually named authors of this work) are: Marilyn Benham, Sagair Hussain, Brian Kirby, Linda Lawson, Kathleen McElhone, Anthony Ormerod, and Caroline Owen.

Members of the PSORT consortium (excluding individually named authors of this work) are: Michael R. Barnes, Paola Di Meglio, Richard Emsley, Andrea Evans, and Katherine Payne.
