# Supplementary material for: Evaluation of a Therapeutic Drug Monitoring Strategy for Adalimumab in Psoriasis: A Prospective Pharmacokinetic‐Pharmacodynamic Study
Source: Clin Transl Sci. 2026 Apr 30;19(5):e70563. doi: 10.1111/cts.70563 (PMC13129494; doi:10.1111/cts.70563)
Supplement: Supplementary file 1 — Figure S1: Goodness‐of‐fit diagnostics for the final adalimumab PK–PD model. (A) Observed versus individual predictions (IPRED) for adalimumab serum concentrations (left) and PASI response (right). (B) Individual weighted residuals (IWRES) versus time (left) and versus individual predictions (right) for the PK model (top panels) and the PD model (bottom panels). (C) Observed versus population predictions (PRED) for the PASI response. (D) Observed versus population predictions (PRED) for adalimumab serum concentrations. In all panels, blue dots represent observed values. The solid black line denotes the line of identity, and the red line represents a LOESS‐smoothed trend through the data. Dashed black lines in the IWRES plots indicate the ±2 boundaries. [file CTS-19-e70563-s003.pdf]

Figure S1. Goodness-of-fit diagnostics for the final adalimumab PK–PD model. (A) Observed versus individual predictions (IPRED) for adalimumab serum concentrations (left) and PASI response (right). (B) Individual weighted residuals (IWRES) versus time (left) and versus individual predictions (right) for the PK model (top panels) and the PD model (bottom panels). (C) Observed versus population predictions (PRED) for the PASI response. (D) Observed versus population predictions (PRED) for adalimumab serum concentrations. In all panels, blue dots represent observed values. The solid black line denotes the line of identity, and the red line represents a LOESS-smoothed trend through the data. Dashed black lines in the IWRES plots indicate the ±2 boundaries.

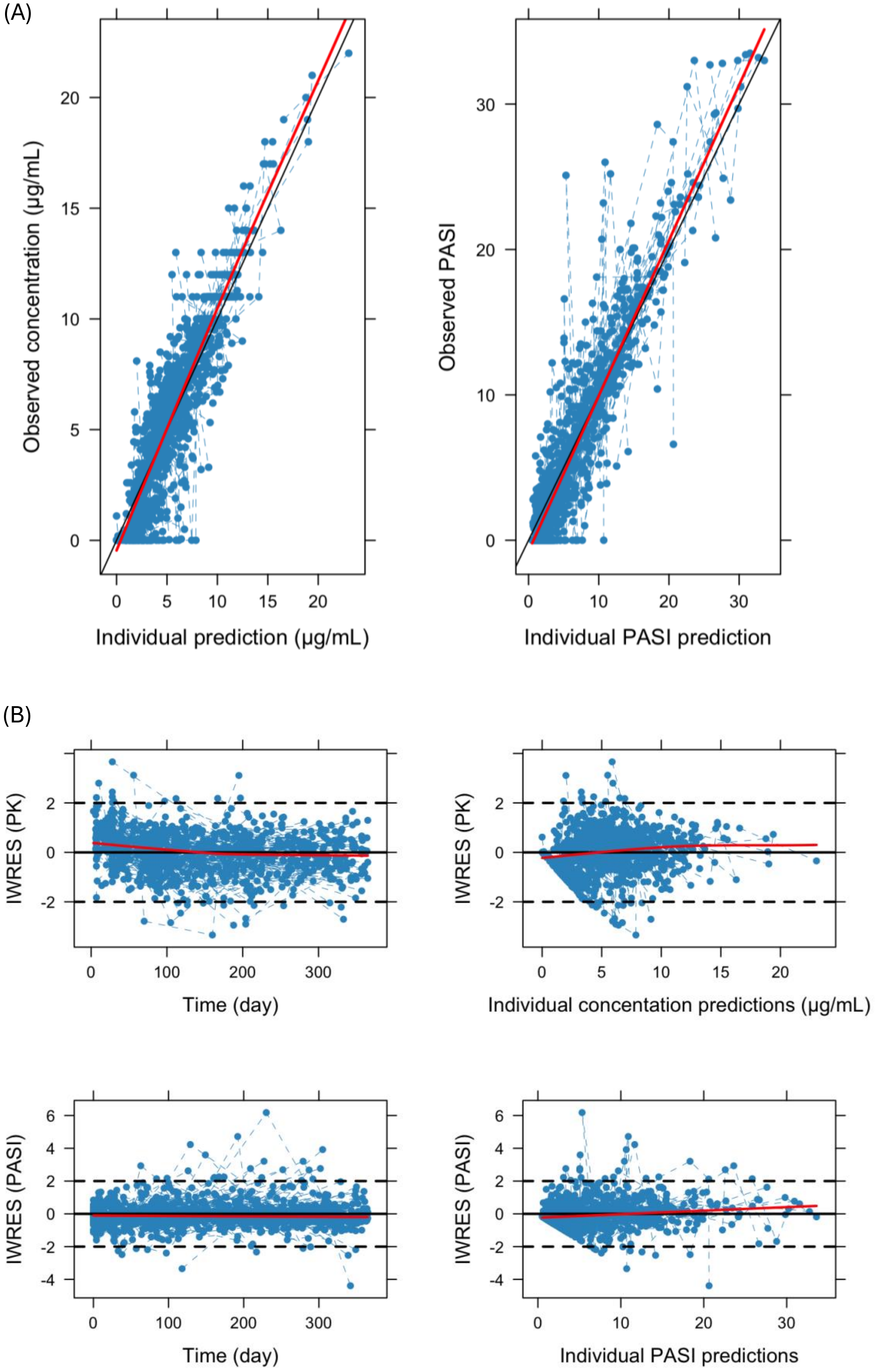

(C)

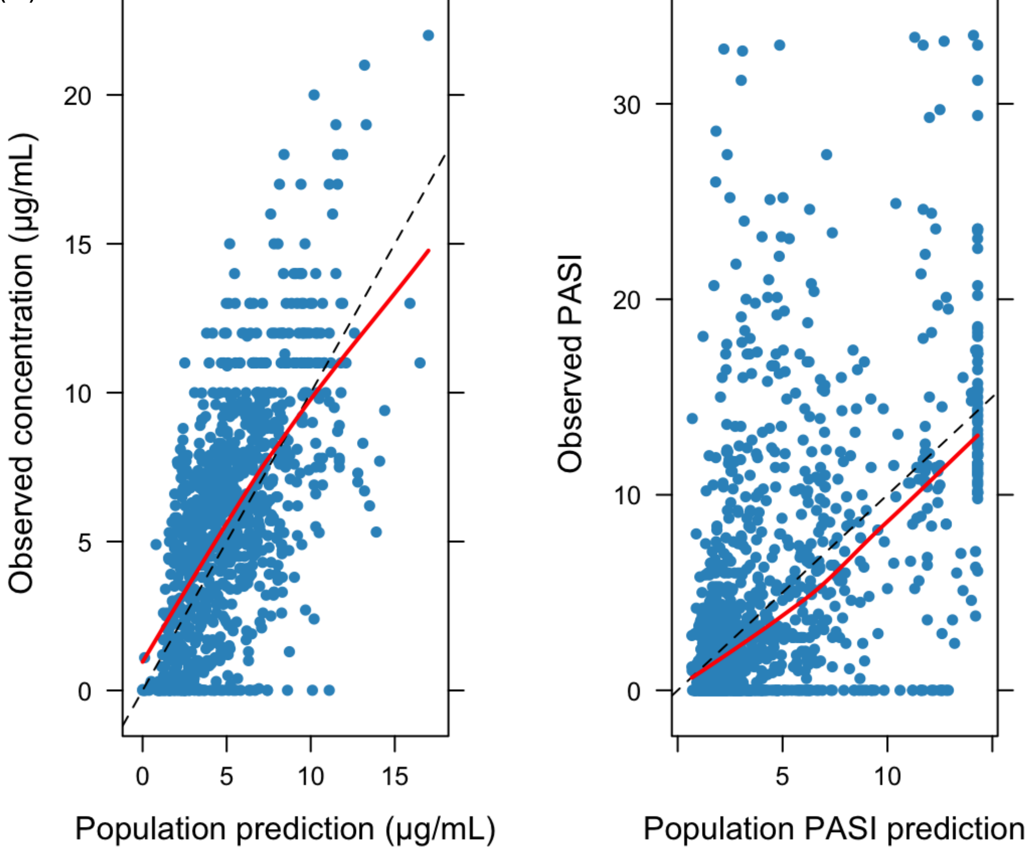
Observed concentration (µg/mL)
Population prediction (µg/mL)
Observed PASI
Population PASI prediction

(D)

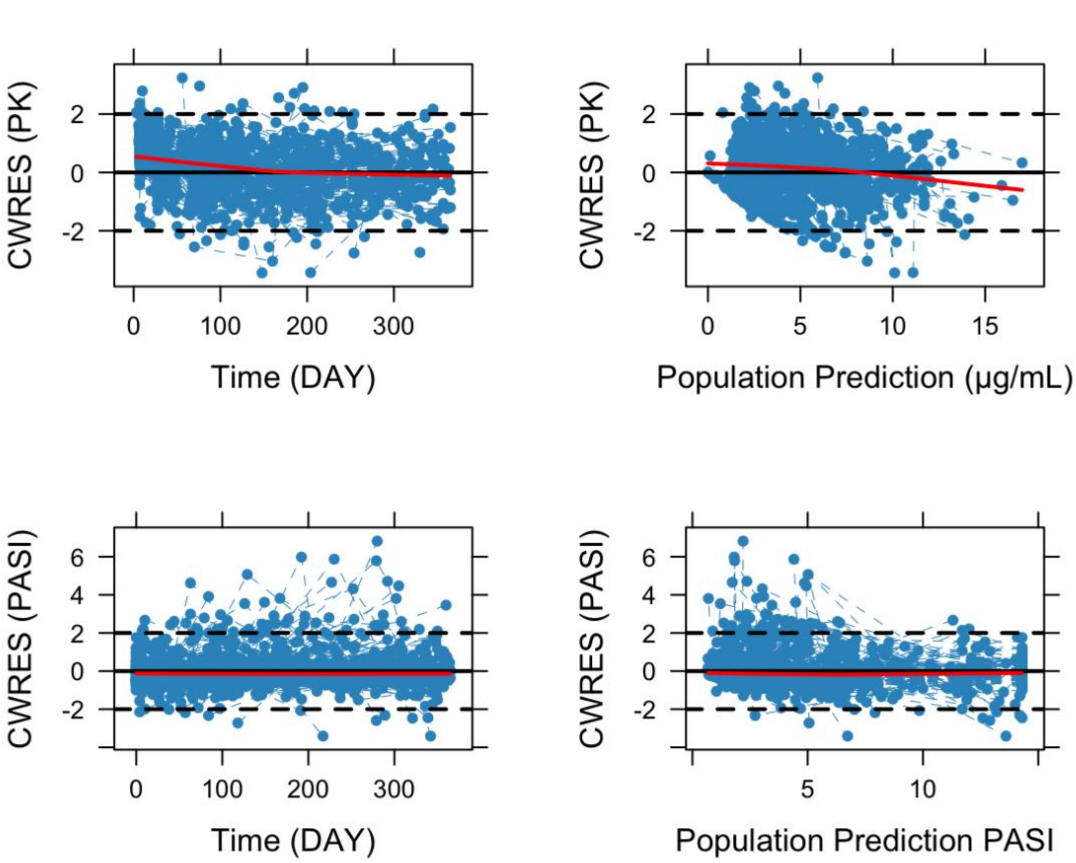
CWRES (PK)
Time (DAY)
CWRES (PK)
Population Prediction (µg/mL)
CWRES (PASI)
Time (DAY)
CWRES (PASI)
Population Prediction PASI
