# Supplementary material for: Evaluation of a Therapeutic Drug Monitoring Strategy for Adalimumab in Psoriasis: A Prospective Pharmacokinetic‐Pharmacodynamic Study
Source: Clin Transl Sci. 2026 Apr 30;19(5):e70563. doi: 10.1111/cts.70563 (PMC13129494; doi:10.1111/cts.70563)
Supplement: Supplementary file 2 — Figure S2: Visual Predictive Checks (VPCs) comparison for (Left) the final one‐compartment PK model, (Middle) the two‐compartment PK model using priors from Kang et al. (2020), and (Right) the two‐compartment PK model using priors from Bobadilla et al. (2023). The solid black line represents the observed median, the dashed black lines denote the observed 5th and 95th percentiles, and the shaded gray areas indicate the simulated 95% prediction interval. The blue band represents the 95% confidence interval around the simulated median. [file CTS-19-e70563-s001.pdf]

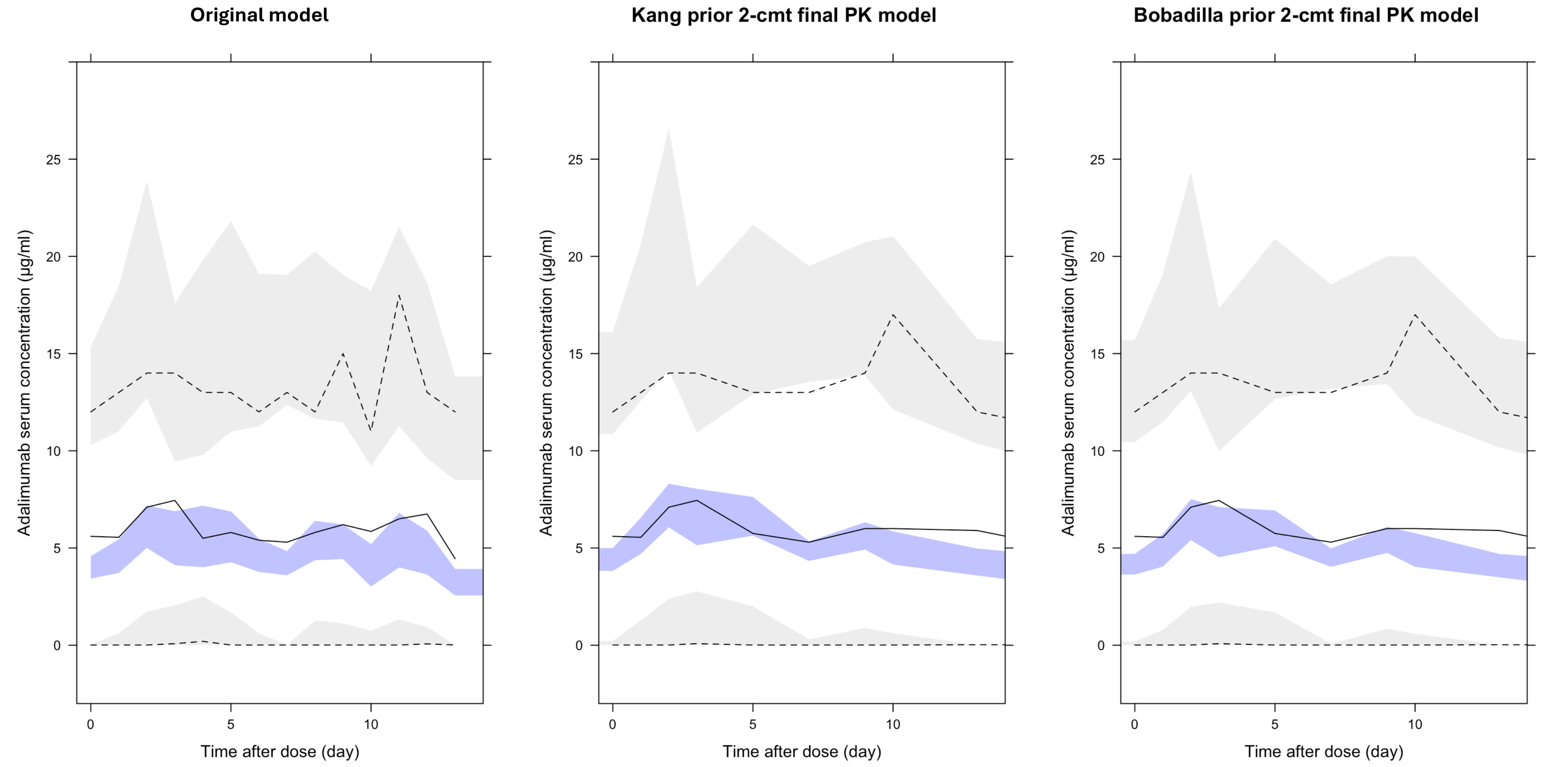

Original model
Kang prior 2-cmt final PK model
Bobadilla prior 2-cmt final PK model
Adalimumab serum concentration (µg/ml)
Time after dose (day)
0
5
10
15
20
25

Figure S2: Visual Predictive Checks (VPCs) comparison for (Left) the final one-compartment PK model, (Middle) the two-compartment PK model using priors from Kang et al. (2020), and (Right) the two-compartment PK model using priors from Bobadilla et al. (2023). The solid black line represents the observed median, the dashed black lines denote the observed 5th and 95th percentiles, and the shaded grey areas indicate the simulated 95% prediction interval. The blue band represents the 95 % confidence interval around the simulated median.
