# Supplementary material for: Evaluation of a Therapeutic Drug Monitoring Strategy for Adalimumab in Psoriasis: A Prospective Pharmacokinetic‐Pharmacodynamic Study
Source: Clin Transl Sci. 2026 Apr 30;19(5):e70563. doi: 10.1111/cts.70563 (PMC13129494; doi:10.1111/cts.70563)
Supplement: Supplementary file 3 — Figure S3: Visual Predictive Checks (VPCs) comparison for the PKPD models describing PASI response over time from (Left) the final one‐compartment PK model, (Middle) the two‐compartment PK model using priors from Kang et al. (2020), and (Right) the two‐compartment PK model using priors from Bobadilla et al. (2023). The solid black line represents the observed median, the dashed black lines denote the observed 5th and 95th percentiles, and the shaded gray areas indicate the simulated 95% prediction interval. The blue band represents the 95% confidence interval around the simulated median. [file CTS-19-e70563-s002.pdf]

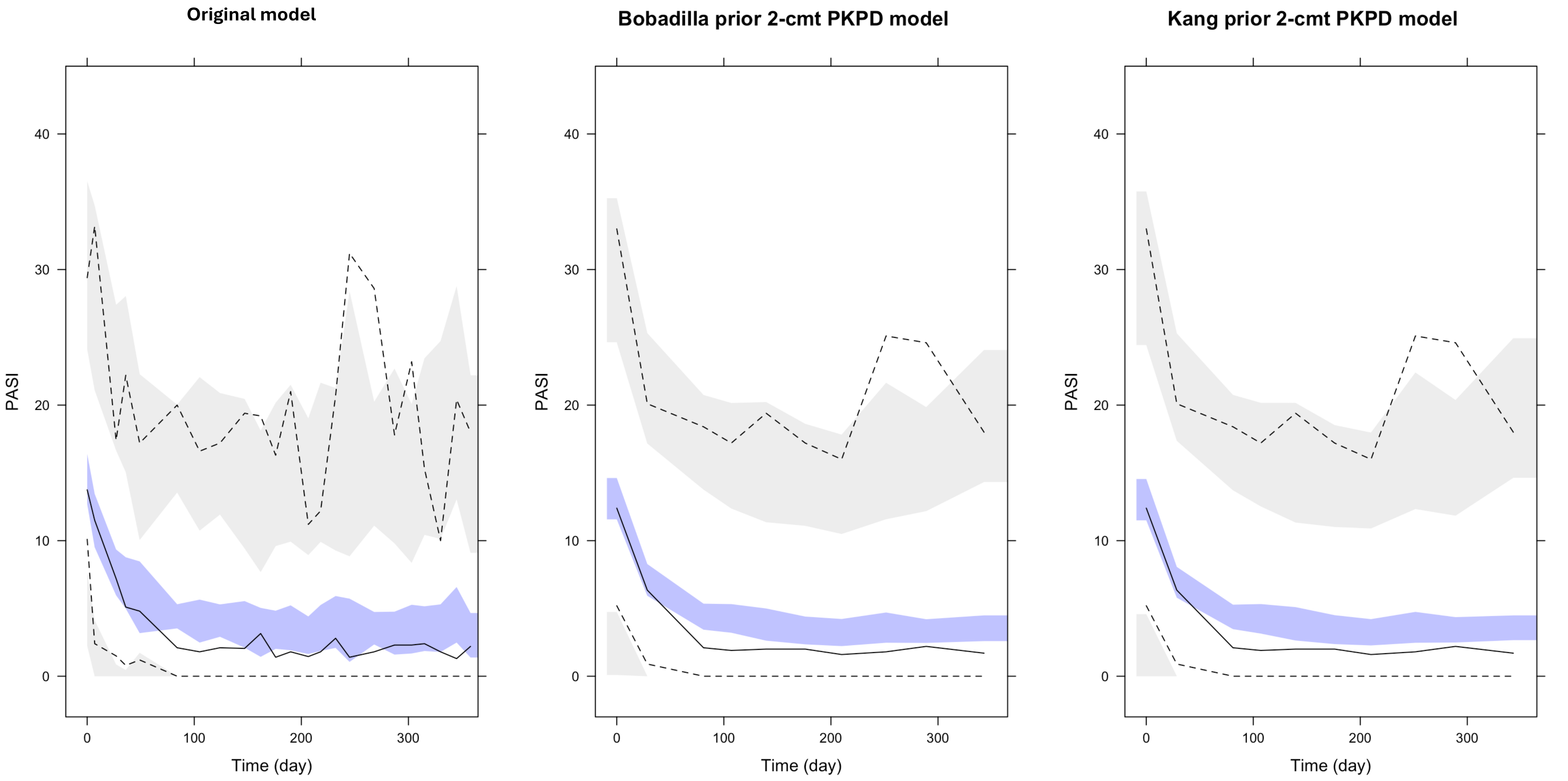
Original model
Bobadilla prior 2-cmt PKPD model
Kang prior 2-cmt PKPD model
PASI
Time (day)
0
10
20
30
40
0
100
200
300

Figure S3: Visual Predictive Checks (VPCs) comparison for the PKPD models describing PASI response over time from (Left) the final one-compartment PK model, (Middle) the two-compartment PK model using priors from Kang et al. (2020), and (Right) the two-compartment PK model using priors from Bobadilla et al. (2023). The solid black line represents the observed median, the dashed black lines denote the observed 5th and 95th percentiles, and the shaded grey areas indicate the simulated 95% prediction interval. The blue band represents the 95 % confidence interval around the simulated median.
