# Supplementary material for: Evaluation of a Therapeutic Drug Monitoring Strategy for Adalimumab in Psoriasis: A Prospective Pharmacokinetic‐Pharmacodynamic Study
Source: Clin Transl Sci. 2026 Apr 30;19(5):e70563. doi: 10.1111/cts.70563 (PMC13129494; doi:10.1111/cts.70563)
Supplement: Supplementary file 4 — Table S1: Parameter estimates from the adalimumab PD model for all patients (n = 539). [file CTS-19-e70563-s005.docx]

Table S1: Parameter estimates from the adalimumab PD model for all patients (n=539).

| **Parameter (unit)** | **Estimate** |  | **RSE (%)** |
| --- | --- | --- | --- |
| baseline PASI | 12.7 |  | 4.8 |
| kout (/day) | 0.04 |  | 20.3 |
| Emax | 1 [fix] |  | - |
| EC50 (μg/mL) | 1.2 |  | 12.7 |
| BSV on baseline (%) | 42.2 |  | 13.0 |
| BSV on kout (%) | 117 |  | 44.8 |
| BSV on EC50 (%) | 94.8 |  | 33.9 |
| Additive error (SD) | 3.2 |  | 6.7 |

PASI: Psoriasis Area Severity Index, PD: pharmacodynamic, k_out_: elimination rate constant of skin lesions, Emax: maximum inhibition effect of adalimumab, EC50: concentration at 50% of maximum inhibition on TNF-α, BSV: between-subject variability, RSE: relative standard error, SD: standard deviation
