# Supplementary material for: Evaluation of a Therapeutic Drug Monitoring Strategy for Adalimumab in Psoriasis: A Prospective Pharmacokinetic‐Pharmacodynamic Study
Source: Clin Transl Sci. 2026 Apr 30;19(5):e70563. doi: 10.1111/cts.70563 (PMC13129494; doi:10.1111/cts.70563)
Supplement: Supplementary file 5 — Table S2: Response rates for subgroups after therapeutic drug monitoring (TDM) dosing or standard‐of‐care (SoC) dosing. [file CTS-19-e70563-s008.docx]

Table S2: Response rates for subgroups after therapeutic drug monitoring (TDM) dosing or standard-of-care (SoC) dosing.

| **Subgroup** | | **1** | **2** | **3** | **4** | **5** | **6** | **Total** |
| --- | --- | --- | --- | --- | --- | --- | --- | --- |
| **Number of patients** | | 83 | 262 | 87 | 104 | 287 | 167 | 990 |
| **TDM dosing** | % PASI75  (6 months) | 100 | 48.9 | 100 | 100 | 64.8 | 65.3 | 70.4 |
|  | % PASI90  (6 months) | 100 | 7.6 | 100 | 86.5 | 27.5 | 15.6 | 38.9 |
| **SoC dosing** | % PASI75  (6 months) | 100 | 39.0 | 100 | 100 | 57.7 | 65.3 | 62.4 |
|  | % PASI90  (6 months) | 100.0 | 6.7 | 100 | 100 | 5.4 | 15.6 | 28.3 |

**Subgroup 1**: trough level concentration < 3.2 μg/mL at week 5 and achieving PASI90 at week 17; **Subgroup 2**: trough level concentration < 3.2 μg/mL at week 5 and NOT achieving PASI90 at week 17; **Subgroup 3**: trough level concentration ≥ 3.2 μg/mL at week 5 and achieving PASI90 at week 17 and trough level concentration ≤ 7 μg/mL at week 17; **Subgroup 4**: trough level concentration ≥ 3.2 μg/mL at week 5 and achieving PASI90 at week 17 and trough level concentration > 7 μg/mL at week 17; **Subgroup 5**: trough level concentration ≥ 3.2 μg/mL at week 5 and NOT achieving PASI90 at week 17 and trough level concentration ≤ 7 μg/mL at week 17; **Subgroup 6**: trough level concentration ≥ 3.2 μg/mL at week 5 and NOT achieving PASI90 at week 17 and trough level concentration > 7 μg/mL at week 17 (PASI: Psoriasis Area Severity Index)
