# Supplementary material for: Evaluation of a Therapeutic Drug Monitoring Strategy for Adalimumab in Psoriasis: A Prospective Pharmacokinetic‐Pharmacodynamic Study
Source: Clin Transl Sci. 2026 Apr 30;19(5):e70563. doi: 10.1111/cts.70563 (PMC13129494; doi:10.1111/cts.70563)
Supplement: Supplementary file 6 — Table S3: Comparison of PK parameter estimates for adalimumab obtained using the final one‐compartment PK model and two two‐compartment PK models implemented with informative Bayesian priors from Kang et al. (2020) [1] and Bobadilla et al. (2023) [2]. [file CTS-19-e70563-s007.docx]

Table S3: Comparison of PK parameter estimates for adalimumab obtained using the final one-compartment PK model and two two-compartment PK models implemented with informative Bayesian priors from Kang et al. (2020) [1] and Bobadilla et al. (2023) [2].

| **PK Parameters (%RSE)** | **Our final model** | **Kang et al. prior** | **Bobadilla et al. prior** |
| --- | --- | --- | --- |
| CL/F | 0.386 (3.5%) | 0.379 (3.3%) | 0.368 (3.1%) |
| Vc/F | 10.8 [fix] | 2.42 (5.8%) | 2.86 (5.5%) |
| Q/F | - | 1.82 (12.7%) | 0.357 (5.6%) |
| Vp/F | - | 3.60 (2.2%) | 4.44 (2.0%) |
| Ka | 0.268 (11.7%) | 0.244 (3.3%) | 0.124 [fix] |
| D1 | - | 0.123 [fix] | - |
| BSV on CL /F(%) | 32.9 (17.9%) | 36.6 (8.6%) | 33.3 (15.5%) |
| BSV on Vc/F (%) | 76.8 (16.5%) | 85.4 (3.7%) | 83.3 (101%) |
| BSV on Q (%) | - | 130.3 (11.5%) | 104.9 (72.9%) |
| BSV on Vq/F (%) | - | 36.3 (11.6%) | 138.4 (70.7%) |
| BSV on Ka (%) | - | 64.4 (11.6%) | - |
| Weight on CL/Q | 0.75 [fix] | 0.75 [fix] CL & Q | 0.832 (CL) [fix] |
| Weight on Vc/Vp | 1 [fix] | 1 [fix] V2 & V3 | 1.83 (Vc) [fix] |
| ADA on CL | 0.368 (6.6%) | 0.366 (5.4%) | 0.382 (5.5%) |
| Female on CL | 0.216 (25.1%) | 0.230 (22.8%) | 0.241 (21.2%) |
| Waist on CL | 0.888 (18.2%) | 0.741 (20.2%) | 0.721 (20.9%) |
| Hypertension on CL | 0.177 (35.1%) | 0.175 (35%) | 0.184 (32.4%) |
| Proportional error % | 19.5 (25.9%) | 15.9 (38.4%) | 17.5 (35.7%) |
| Additive error (SD) | 1.78 (8.8%) | 1.84 (11.3%) | 1.8 (13.4%) |

PK: pharmacokinetic, CL/F: apparent clearance, Vc/F: apparent central volume, Q/F: apparent inter-compartmental clearance, Vp/F: apparent peripheral volume, ka: first-order absorption rate, D1: 0-order absorption rate, BSV: between-subject variability, ADA: anti-drug antibody, RSE: relative standard error, SD: standard deviation

**Reference:**

[1] Kang J, Eudy-Byrne RJ, Mondick J, Knebel W, Jayadeva G, Liesenfeld KH. Population pharmacokinetics of adalimumab biosimilar adalimumab-adbm and reference product in healthy subjects and patients with rheumatoid arthritis to assess pharmacokinetic similarity. Br J Clin Pharmacol. 2020 Nov;86(11):2274-2285. doi: 10.1111/bcp.14330. Epub 2020 Jun 11. PMID: 32363771; PMCID: PMC7576631.

[2] Ponce-Bobadilla, A.V., Stodtmann, S., Chen, MJ. et al. Assessing the Impact of Immunogenicity and Improving Prediction of Trough Concentrations: Population Pharmacokinetic Modeling of Adalimumab in Patients with Crohn’s Disease and Ulcerative Colitis. Clin Pharmacokinet 62, 623–634 (2023). https://doi.org/10.1007/s40262-023-01221-x
