# Supplementary material for: Evaluation of a Therapeutic Drug Monitoring Strategy for Adalimumab in Psoriasis: A Prospective Pharmacokinetic‐Pharmacodynamic Study
Source: Clin Transl Sci. 2026 Apr 30;19(5):e70563. doi: 10.1111/cts.70563 (PMC13129494; doi:10.1111/cts.70563)
Supplement: Supplementary file 7 — Table S4: Comparison of PD parameter estimates from the final one‐compartment PKPD model and those from two‐compartment PK models using Kang et al. and Bobadilla et al. priors; the relative percentage change is calculated relative to the final one‐compartment model. [file CTS-19-e70563-s006.docx]

Table S4: Comparison of PD parameter estimates from the final one-compartment PKPD model and those from two-compartment PK models using Kang et al. and Bobadilla et al. priors; the relative percentage change is calculated relative to the final one-compartment model.

| **PD Parameters (%RSE)** | **Our final model** | **Kang et al. prior** | **% Change** | **Bobadilla et al. prior** | **% Change** |
| --- | --- | --- | --- | --- | --- |
| Baseline PASI (BSL) | 14.3 (4.6%) | 14.2 (3.8%) | -0.7% | 14.3 (4.3%) | 0% |
| Kout | 0.04 (7.7%) | 0.0368 (9.2%) | -8% | 0.039 (9.4%) | -2.5% |
| Emax | 1 [fix] | 1 [fix] | - | 1 [fix] | - |
| IC50 | 0.95 (13.7%) | 0.955 (13.0%) | -0.53% | 0.946 (14.5%) | -0.42% |
| BSV on BSL (%) | 38.6 (16.0%) | 40.9 (16.8%) | +5.96% | 40.0 (15.8%) | +3.63% |
| BSV on Kout (%) | 116.6 (19.7%) | 161.1 (18.7%) | +38.16% | 164.5 (21.6%) | +41.08% |
| BSV on IC50 (%) | 97.1 (22.0%) | 124.2 (22.9%) | +27.91% | 131.0 (22.8%) | +34.91% |
| Additive error (SD) | 3.2 (7.8%) | 3.22 (15.3%) | +0.63% | 3.24 (15.4%) | +1.25% |

PASI: Psoriasis Area Severity Index, PD: pharmacodynamic, k_out_: elimination rate constant of skin lesions, Emax: maximum inhibition effect of adalimumab, IC50: concentration at 50% of maximum inhibition on TNF-α, BSV: between-subject variability, RSE: relative standard error, SD: standard deviation.

**Reference:**

[1] Kang J, Eudy-Byrne RJ, Mondick J, Knebel W, Jayadeva G, Liesenfeld KH. Population pharmacokinetics of adalimumab biosimilar adalimumab-adbm and reference product in healthy subjects and patients with rheumatoid arthritis to assess pharmacokinetic similarity. Br J Clin Pharmacol. 2020 Nov;86(11):2274-2285. doi: 10.1111/bcp.14330. Epub 2020 Jun 11. PMID: 32363771; PMCID: PMC7576631.

[2] Ponce-Bobadilla, A.V., Stodtmann, S., Chen, MJ. et al. Assessing the Impact of Immunogenicity and Improving Prediction of Trough Concentrations: Population Pharmacokinetic Modeling of Adalimumab in Patients with Crohn’s Disease and Ulcerative Colitis. Clin Pharmacokinet 62, 623–634 (2023). https://doi.org/10.1007/s40262-023-01221-x
