# Supplementary material for: Evaluation of a Therapeutic Drug Monitoring Strategy for Adalimumab in Psoriasis: A Prospective Pharmacokinetic‐Pharmacodynamic Study
Source: Clin Transl Sci. 2026 Apr 30;19(5):e70563. doi: 10.1111/cts.70563 (PMC13129494; doi:10.1111/cts.70563)
Supplement: Supplementary file 8 — Data S1: Supporting Information. [file CTS-19-e70563-s004.docx]

**Code S1: NONMEM code for the PK model of adalimumab**

$PROBLEM ADALIMUMAB 1-CPT PK MODEL

$INPUT ID AMT=DOSE TIME=DAY EVID DVID DV MDV ANTIADL ARIA BL_PASI

GENDER ETHNICITY=DROP PALMS=DROP HEIGHT WEIGHT WAIST

BMI=DROP AGE=DROP DURATION CREATININE=DROP BIO_NAIVE=DROP

ANY_IA=DROP ALCO=DROP SMOK DIAB=DROP HYPE DEPR=DROP

ASTH=DROP DYSL=DROP LIVE=DROP CENSORANTIADL_IDV CENSORARIA_IDV

$DATA ADA_NMPK_v3.csv IGNORE=@

$SUBROUTINE ADVAN2 TRANS2

$PK

;;; CLWAIST-DEFINITION START

IF(WAIST.EQ.-99) THEN

CLWAIST = 1

ELSE

CLWAIST = ((WAIST/101)**THETA(7))

ENDIF

;;; CLWAIST-DEFINITION END

;;; CLHYPE-DEFINITION START

IF(HYPE.EQ.0) CLHYPE = 1 ; Most common

IF(HYPE.EQ.1) CLHYPE = ( 1 + THETA(6))

;;; CLHYPE-DEFINITION END

;;; CLGENDER-DEFINITION START

IF(GENDER.EQ.1) CLGENDER = 1 ; Most common

IF(GENDER.EQ.0) CLGENDER = ( 1 + THETA(5))

;;; CLGENDER-DEFINITION END

;;; CLARIA-DEFINITION START

CLARIA = ((ARIA/76.03)**THETA(4))

;;; CLARIA-DEFINITION END

;;; CL-RELATION START

CLCOV=CLARIA*CLGENDER*CLHYPE*CLWAIST

;;; CL-RELATION END

F1 = 1

KA = THETA(1)

TVCL = THETA(2)*(WEIGHT/70)**0.75

TVCL = CLCOV*TVCL

CL = TVCL*EXP(ETA(1))

TVV = THETA(3)*(WEIGHT/70)**1

V = TVV*EXP(ETA(2))

$ERROR

IPRED = A(2)/V

Y = IPRED*(1 + EPS(1)) + EPS(2)

PROP = SQRT(SIGMA(1,1))*IPRED

ADD = SQRT(SIGMA(2,2))

SD = SQRT(PROP*PROP + ADD*ADD)

IRES = DV-IPRED

IWRES = IRES/SD

;--- REPLACE LOW DV VALUES

LOQ = 0.01

TDV = DV

IF(TDV<LOQ) TDV = LOQ

;--- TIME AFTER DOSE

IF(EVID==1) TM=TIME

IF(EVID==1) TAD=0

IF(EVID/=1) TAD=TIME-TM

$THETA

(0,0.272138) ; KA (/day)

(0,0.361449) ; CL (L/day)

10.8 FIX ; V (L)

$THETA (-100,0.362013,100000) ; CLARIA1

$THETA (-1,0.198852,5) ; CLGENDER1

$THETA (-1,0.226482,5) ; CLHYPE1

$THETA (-100,0.771436,100000) ; CLWAIST1

$OMEGA

0.111545

0.421423

$SIGMA

0.1

0.1

$ESTIMATION MAXEVAL=9999 METHOD=1 INTERACTION POSTHOC NOABORT PRINT=5

$COVARIANCE UNCONDITIONAL PRINT=E

$TABLE ID TIME AMT DVID PRED IPRED CWRES TAD NOPRINT ONEHEADER FILE=sdtab17

$TABLE ID KA CL V ETA1 ETA2 NOPRINT NOAPPEND ONEHEADER FILE=patab17

$TABLE ID CENSORARIA_IDV NOPRINT NOAPPEND ONEHEADER FILE=cotab17

$TABLE ID NOPRINT NOPRINT NOAPPEND ONEHEADER FILE=catab17

**Code S2: NONMEM code for the PKPD model of adalimumab**

$PROBLEM ADALIMUMAB PKPD TURNOVER WITH IPP

$INPUT ID AMT=DOSE TIME=DAY EVID DVID DV MDV ANTIADL ARIA BL_PASI GENDER ETHNICITY PALMS HEIGHT WEIGHT WAIST BMI AGE DURATION CREATININE BIO_NAIVE ANY_IA ALCO SMOK DIAB HYPE DEPR ASTH DYSL LIVE CENSORANTIADL_IDV CENSORARIA_IDV IKA ICL IV

$DATA ADA_NMPKPD_v2.csv IGNORE=@ IGNORE=(DVID.EQ.1, DVID.GT.2) IGNORE=(BL_PASI<10) IGNORE=(ID.EQ.142)

$SUBROUTINE ADVAN6 TOL=6

$MODEL

COMP=(DOSE)

COMP=(CENTRAL)

COMP=(PASI)

$PK

F1 = 1

KA = IKA

CL = ICL

V = IV

KE = CL/V

BSL = THETA(1)*EXP(ETA(1))

TVKOUT = THETA(2)

KOUT = TVKOUT*EXP(ETA(2))

EMAX = THETA(3)

TVIC50 = THETA(4)

IC50 = TVIC50*EXP(ETA(3))

KIN = BSL*KOUT

A_0(3) = BSL

TDV=DV

IF(TDV<=0) TDV=0.01

;--- Time after dose

IF(EVID==1) TM=TIME

IF(EVID==1) TAD=0

IF(EVID/=1) TAD=TIME-TM

$DES

DADT(1) = -KA*A(1)

DADT(2) = KA*A(1) - KE*A(2)

CONC = A(2)/V

DG = EMAX*CONC/(IC50+CONC)

DADT(3) = KIN*(1-DG)-KOUT*A(3)

$ERROR

IPRED = A(3)

Y = IPRED + EPS(1)

ADD = SQRT(SIGMA(1,1))

SD = ADD

IRES = DV-IPRED

IWRES = IRES/SD

$THETA

(0, 15) ;BSL

(0, 0.05) ;KOUT (per DAY)

1 FIX ;EMAX

(0, 0.5) ;IC50 (ug/mL)

$OMEGA

0.1

0.1

0.1

$SIGMA

0.1

$ESTIMATION MAXEVAL=9999 METHOD=1 INTERACTION POSTHOC NOABORT PRINT=5

$COV UNCONDITIONAL PRINT=E

$TABLE ID TIME DVID IPRED IWRES CWRES TAD EVID NOPRINT ONEHEADER FILE=sdtab3

$TABLE ID BSL KOUT EMAX IC50 ETA1 ETA2 ETA3 NOPRINT NOAPPEND ONEHEADER FILE=patab3

$TABLE ID NOPRINT NOAPPEND ONEHEADER FILE=cotab3

$TABLE ID NOPRINT NOAPPEND ONEHEADER FILE=catab3
